# Fine-scale harbour seal usage for informed marine spatial planning

**DOI:** 10.1038/s41598-017-11174-4

**Published:** 2017-09-14

**Authors:** Esther L. Jones, Carol E. Sparling, Bernie J. McConnell, Christopher D. Morris, Sophie Smout

**Affiliations:** 1 Centre for Research into Ecological and Environmental Modelling, The Observatory, Buchanan Gardens, University of St Andrews, St Andrews, Fife KY16 9LZ UK; 2 Sea Mammal Research Unit, Scottish Oceans Institute, University of St Andrews, St Andrews, Fife KY16 8LB UK; 3 SMRU Consulting Ltd, New Technology Centre, University of St Andrews, North Haugh, St Andrews, Fife KY16 9SR UK

## Abstract

High-resolution distribution maps can help inform conservation measures for protected species; including where any impacts of proposed commercial developments overlap the range of focal species. Around Orkney, northern Scotland, UK, the harbour seal (*Phoca vitulina*) population has decreased by 78% over 20 years. Concern for the declining harbour seal population has led to constraints being placed on tidal energy generation developments. For this study area, telemetry data from 54 animals tagged between 2003 and 2015 were used to produce density estimation maps. Predictive habitat models using GAM-GEEs provided robust predictions in areas where telemetry data were absent, and were combined with density estimation maps, and then scaled to population levels using August terrestrial counts between 2008 and 2015, to produce harbour seal usage maps with confidence intervals around Orkney and the North coast of Scotland. The selected habitat model showed that distance from haul out, proportion of sand in seabed sediment, and annual mean power were important predictors of space use. Fine-scale usage maps can be used in consenting and licensing of anthropogenic developments to determine local abundance. When quantifying commercial impacts through changes to species distributions, usage maps can be spatially explicitly linked to individual-based models to inform predicted movement and behaviour.

## Introduction

Within the context of increasing anthropogenic activities in coastal environments, understanding movement and distributions of top predators is critical to deliver effective marine spatial planning and ensure adequate management and protection^1–4^. However, marine animals are challenging to study as they spend all or most of their lives at sea, and much of this time underwater. Robust estimates of space use at appropriate spatial and temporal scales are required and should include estimates of uncertainty to ensure that risks to the population can be identified and managed objectively.

In early-stage marine spatial planning, constraint mapping is carried out to reduce conflicts and ensure sustainable use of marine resources. For example, areas are identified for potential commercial development, such as defining lease areas for proposed offshore marine renewable projects, whilst ensuring the conservation of protected species and habitats (e.g. marine protected areas; www.gov.uk/government/publications/east-inshore-and-east-offshore-marine-plans). During consenting and licensing stages, a common approach is to overlay spatial layers within a Geographical Information System (GIS) framework, such as anthropogenic activities and species distributions, so that areas of interest and associated risks can be identified^5^. Anthropogenic activities in the marine environment are often resolved to a fine spatio-temporal scale (e.g. locations of marine energy leasing areas or oil and gas pipelines), and to improve efficacy in marine spatial planning it is important to also use high resolution and robust maps of species distributions and habitats prioritised for conservation. Estimates of uncertainty in species distributions should be generated to inform decision-making regarding the level of identified risks.

Harbour seals (*Phoca vitulina*) are one of two resident seal species around the UK, spending the majority of their time within 50 km of the coast^6^. Around Orkney, their diet (in 2010/11) was dominated by sandeel (*Ammodytes spp*), cod (*Gadus morhua*), and saithe (*Pollachius virens*) in spring and summer, and pelagic and gadid fish (mainly herring (*Clupea harengus*) and cod) in autumn^7^. They haul out for extended periods to breed in June and July, and moult in August^8^. The ‘Habitats Directive’ (1992 Directive on the Conservation of Natural Habitats and of Wild Fauna and Flora (92/43/EEC)), is one of the main policy drivers for nature conservation in European waters including the UK. The Habitats Directive is transposed into Scottish law by the Conservation (Natural Habitats) Regulations 1994 (as amended in Scotland), and under these Regulations Special Areas of Conservation (SACs) have been established for harbour seals. The harbour seal population has been in decline in some areas around the UK since at least 2000. Animals within the Orkney and the North Coast management region have been particularly affected with numbers decreasing by 78% between 1997 and 2013^9^. Concern around the status of the population, coupled with uncertainty surrounding the risk of collisions between tidal turbines and seals, has led to constraints being placed on tidal energy generation developments in this area until more information is available on the potential risks presented to this species by tidal turbines. A key element of models for assessing collision risk is determining the abundance of animals that may use the area close to the turbines.

Orkney and the North coast of Scotland is an interesting study area: it has a convoluted coastline with diverse physical environment and sediment dynamics, including the Pentland Firth, an area with strong tidal currents^10^. The declining local harbour seal population, coupled with the world’s first commercial tidal stream array (www.meygen.com) now in place, makes characterisation of seal usage at a more appropriate scale for assessing individual project development essential for effective spatial management.

Maps of at-sea usage of harbour seals around Orkney and the North coast of Scotland were produced with associated 95% confidence intervals. Based on established methodology^6^, analytical capabilities were enhanced to address scalability, uncertainty, and predictive power. We implemented an analytical solution with high spatial resolution to more appropriately reflect underlying heterogeneity in seal movement, reduced uncertainty by clustering similar haul outs to ensure underlying telemetry data were retained in the analysis, and incorporated environmental covariates pertinent to the species in a more sophisticated modelling framework to predict space use in regions where telemetry data were unavailable.

## Results

**Figure 1:**
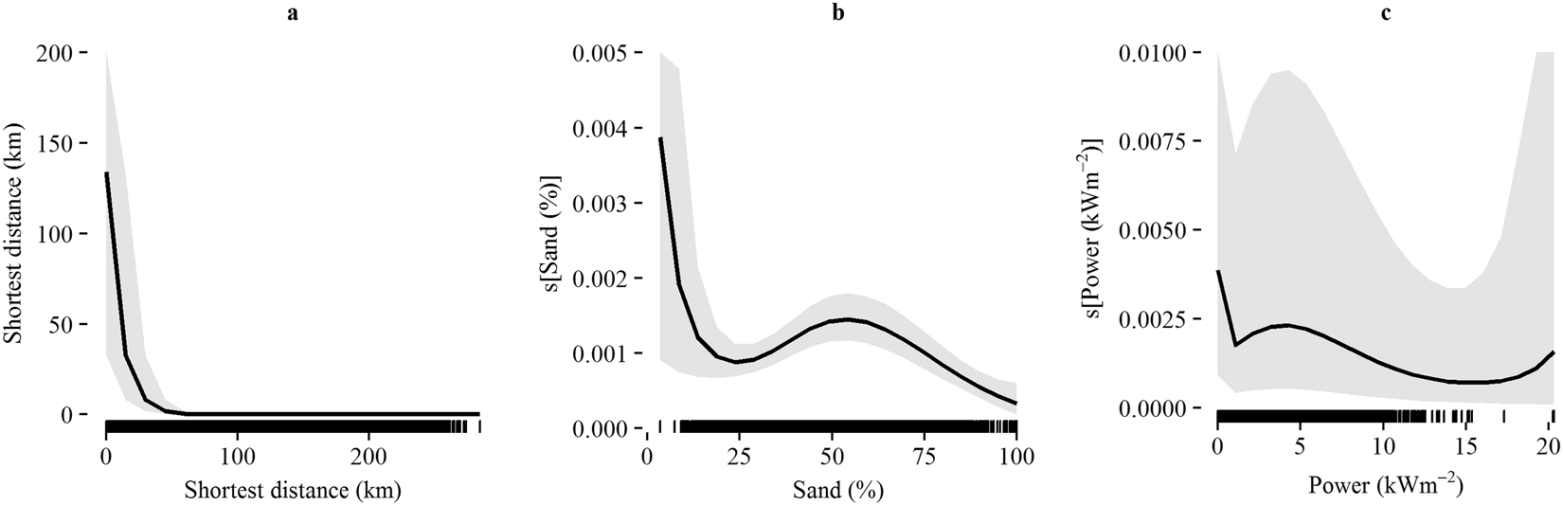
Occurrence rate of animals, predicted by the selected model (i.e. mean population responses) for each covariate (**a**) Shortest at-sea distance to haul out, (**b**) Proportion of sand in sediment, (**c**) Annual mean tidal power. Occurrence rate is calculated on the scale of the exponential of the linear predictor (proportional to usage) (y-axes) with shaded areas representing 95% confidence intervals (using parametric bootstrapping). Rug plots showing data values are displayed on the x-axis of each plot.

Year and shortest at-sea distance from haul out were included in the selected habitat model. Shortest at-sea distance was required so that predicted usage for each null cluster was limited according to the distance that an animal could realistically travel from the cluster. Figure 1 shows the occurrence rate for each covariate (response variable on the scale of the exponential of the linear predictor; y-axes) with accompanying 95% confidence intervals calculated through parametric bootstrapping. As expected, shortest at-sea distance had a strongly negative coefficient, indicating that usage decreased with increasing distance from haul out. Proportion of sand in sediment and annual mean tidal power were retained in the selected model as polynomial terms. High usage of low proportions of sand was associated with wide confidence intervals, as data were limited. Space use then increased with increasing proportion of sand, peaking when sediment was 54% sand. The relationship between usage and annual mean tidal power showed that usage generally decreased with increasing tidal power, although confidence intervals were wide.

**Figure 2:**
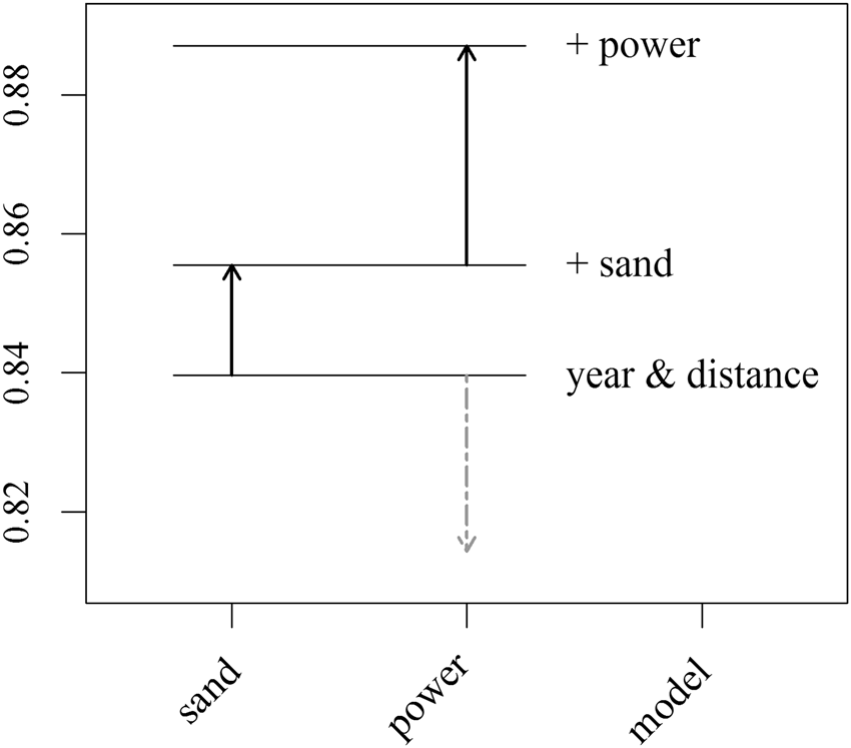
Forwards model selection resulted in increasingly complex models until five-fold cross-validation scores (using 40 equal bins areas) did not improve. The length and direction of the arrows indicate change in cross-validation score following the addition of each covariate. Solid arrows indicate the variables that led to the largest improvement in score.

The relative contribution of each covariate to model selection is shown in Fig. 2. The model with only year and at-sea distance covariates produced a fold pass score (FPS = 0.84), above threshold (0.80) using 40 equal-size bins. Including sand increased the cross-validation score (FPS = 0.86) and including tidal power raised the score (FPS = 0.89). This FPS could not be improved upon with additional covariates. We speculate that the decrease in score when tidal power was added to the baseline model was due to an unquantified interaction between tidal power and at-sea distance. Interactions could not be included in model selection due to non-convergence of the GAM-GEEs.

**Figure 3:**
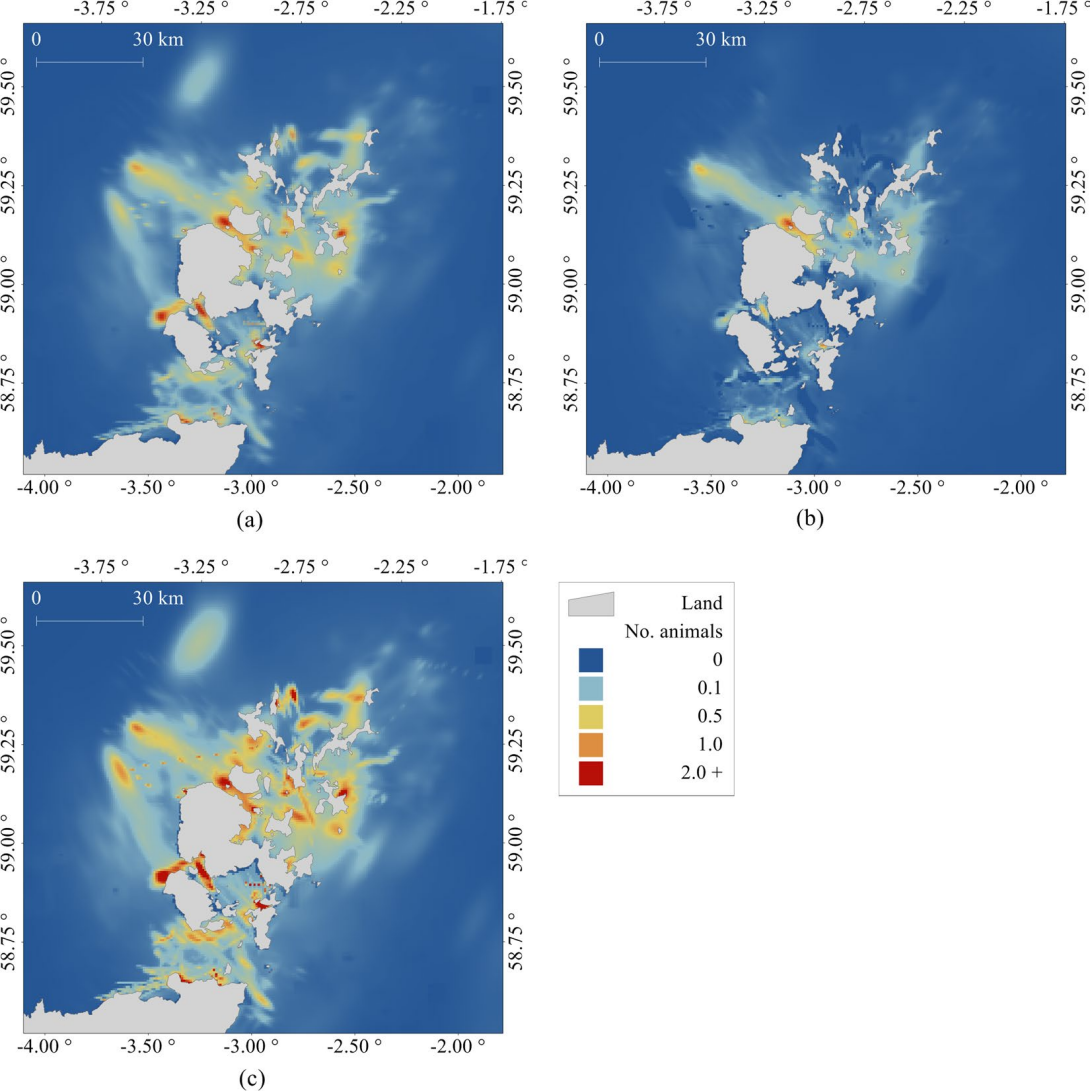
At-sea harbour seal usage (**a**) mean, (**b**) lower 95% confidence interval, (**c**) upper 95% confidence interval. The figure was produced using R 3.3.2^35^ and GIS software Manifold 8.0.29.0^35^.

**Figure 4:**
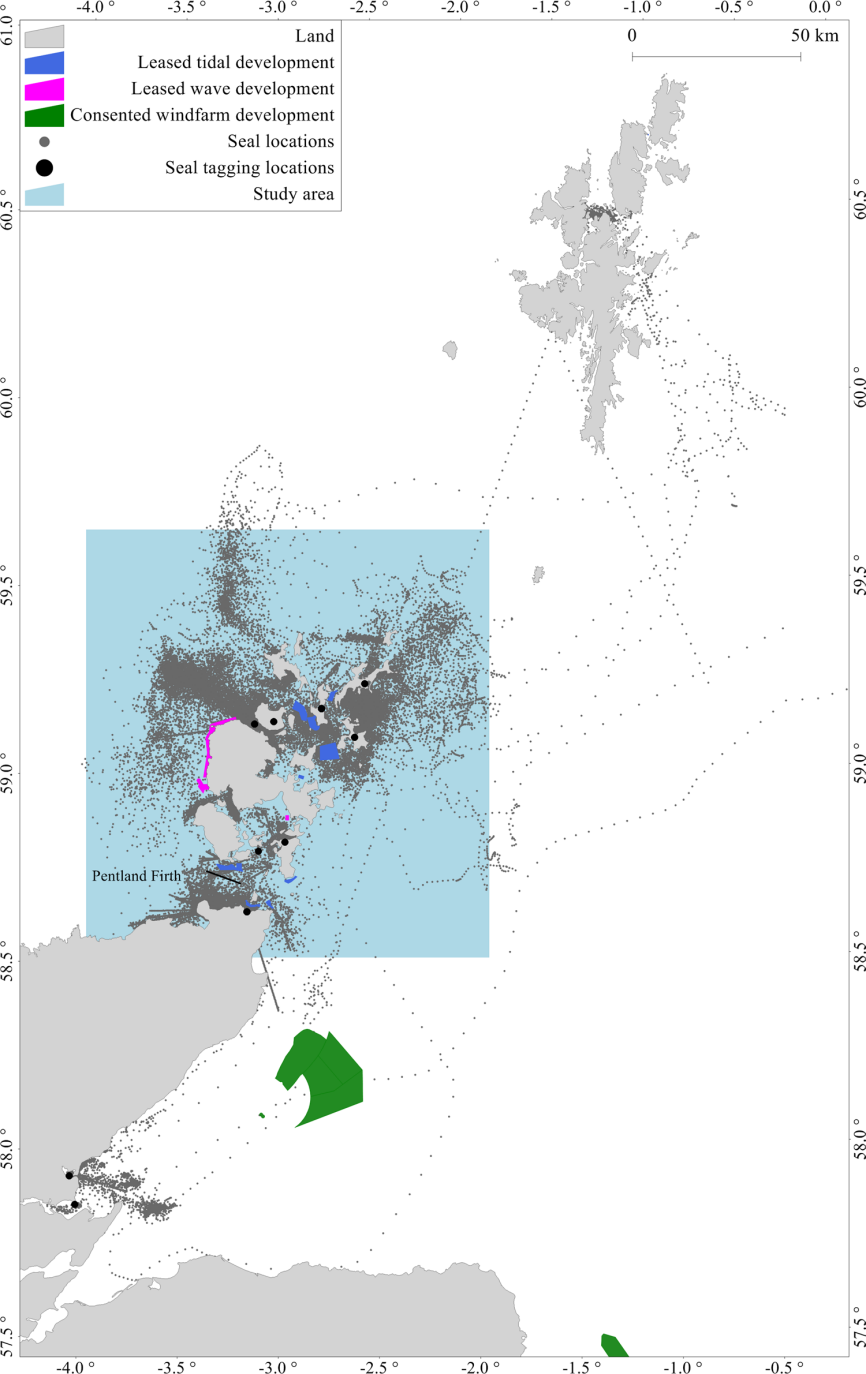
Map showing the spatial extent of the analysis, tracks of 54 animals (dark grey points), their tagging locations (black circles), proposed offshore marine renewable developments (tidal stream (blue), wave (pink), wind (green) areas), and study area centred on Orkney (blue background). The figure was produced using R 3.3.2^34^ and GIS software Manifold 8.0.29.0^35^.

Usage based on telemetry observations comprised 82% and the habitat modelling contributed 18% to the at-sea map. Figure 3a shows at-sea distributions of harbour seals around Orkney and the North coast of Scotland, and can be interpreted as the estimated mean number of seals present in each 0.6 km × 0.6 km cell. The map shows that harbour seals spend the majority of their time within 30 km of the coast around Orkney and the North coast of Scotland, and that much of the centre of the channel of the Pentland Firth is not well utilised (Fig. 4). Figure 3b and c show lower and upper 95% confidence intervals and can be interpreted as the bounds on the estimated number of seals in each cell. Harbour seal at-sea usage across the whole map is estimated as 2444 (95% CI 946, 4006). Aggregating haul outs at 3.6 km gave rise to 246 telemetry clusters (haul out clusters with telemetry data associated with them). Seven of these clusters had only one tagged animal and a terrestrial count greater than one, which contributed to approximately to 7% of the total at-sea mean usage calculated from the maps. 45% of total at-sea usage (over half of the telemetry usage contribution to the maps) arose from data-rich clusters with ≥ 7 tagged animals associated with them (Fig. S3 in Supplementary information). It is important to note that at-sea usage in any given cell is influenced by density estimation from multiple telemetry clusters, and predicted usage from null clusters. Therefore, cases where few tagged animals were explicitly associated with a haul out cluster did not necessarily mean that only usage from these individuals influenced the total usage of that cluster.

## Discussion

Maps of harbour seal at-sea usage were produced for the area around Orkney and the North coast of Scotland, with associated 95% confidence intervals. These high resolution maps with levels of uncertainty around the mean distribution of animals can be used to inform spatial management of the marine environment.

Harbour seals are central-place foragers, regularly hauling out on land in between spending time at sea travelling and foraging. Therefore, their at-sea distribution is likely to be strongly linked to their haul out locations. At-sea usage maps show that harbour seals around Orkney concentrated space use within 30 km of their haul outs, a behaviour consistent with other areas around the UK^6, 11–13^. The primary driver of space use was distance from haul out in the predictive habitat model; usage declined with increasing distance from haul out. More specifically, animals spent the majority of time within a few kilometres of the coast in shallow water (less than 50 m); an exception was to the north-west of the Orkney mainland where animals spent time further offshore presumably foraging at sand banks^14^. From the habitat model, the second driver of seal usage was the proportion of sand in sediment. Excluding the lower range where data were limited, harbour seal usage increased until 54% sand, whereby usage then declined. Sandeels are non-migratory resident species that live in sand/gravel mix sediment^15, 16^. They are a primary component in the diet of harbour seals around Orkney^7^, and the relationship found between seal space use and sand could be driven by this predator-prey interaction. The Pentland Firth, an area with strong tidal currents, is of commercial interest as a number of leased tidal developments are situated within it. Usage within the Pentland Firth itself was limited although haul outs were situated to the north and south of the channel. The relationship between seal usage and annual mean tidal power showed that harbour seal space use generally declined with increasing power. Relationships found between shortest at-sea distance from haul out and seabed sediment corroborate with other literature that have found these relationships in grey seal habitat preference in the North Sea^17^. Like any predator, seals most likely respond dynamically to their environment with regards to the location of their prey species, and sand in sediment and annual mean tidal power are likely to act as proxies for prey distribution^18, 19^. Free-ranging marine animals such as seals must be influenced by currents, either positively by using currents to travel more efficiently and utilise concentrated prey patches, or negatively by swimming against currents when travelling to a haul out, which may vary regionally^19–22^. Relevant environmental covariates were used for habitat selection modelling but other covariates likely to be good predictors of space use could be included. The composition of harbour seal diet is known to change over time and region^7^. Where available, temporally and spatially aligned prey data may be likely candidates to increase predictive ability^23^ (although see^24^).

It is important to understand how species distributions change over time. However, animal location data are usually incomplete across time and space as a result of patchy data collection. The modelling framework was developed to handle partial data, accounting for areas where no data were available (unobserved regions), as well as quantifying the accompanying uncertainty. This methodology could be extended using historical data sets to investigate temporal changes in distribution such as seasonal changes and inter-annual fluctuations, so that long-term changes in abundance and distribution can be captured to inform conservation of the species. Usage maps were scaled to population estimates using terrestrial counts collected during August. During this time, harbour seals moult, spending much of their time hauled out, and the terrestrial counts provide information about the abundance and distribution of seals at this stage in their lifecycle. Intra-annual movement of individuals outside of the study area, or the distribution of animals between haul out sites within the study area were not accounted for. To identify these, and seasonal changes in distribution, additional terrestrial counts outside of moult season would be required, as well as an estimate of proportion of animals hauling out when these additional surveys were carried out^25^.

Usage in a given at-sea grid cell was a complex summation (including weightings) of maps from different haul out clusters. For any given grid cell, there are likely to be substantial contributions from several clusters, and those with few seals associated with them are likely to have low weights. Any given grid cell will be influenced by null maps from clusters where there are no telemetry data. To account for any extreme seal behaviour from one animal at a haul out cluster, each kernel smooth was reweighted by the index of information content (by individual) based on the relative amount of information the animal contributed (tagged hours per animal and tag type). This method ensured that fine-scale features in space use were retained, whilst not emphasizing abnormal behaviour of individuals. Uncertainty in the usage where results from some haul out clusters having few seal trips were influential was represented in the confidence interval maps (i.e. wider confidence intervals in those areas). Usage was displayed over all types of seal activity without distinguishing between habitat that may be important for specific events, such as foraging or breeding, from areas that might be used as ‘commuting corridors’ between such sites. Anthropogenic activities can have chronic impacts on marine species such as avoidance of important habitats, or changes to behaviour^26^. One way to assess these impacts is to quantify the population effects on the species; energetic costs to animals vary by activity^27^ and therefore explicitly accounting for activity budgets would be required. When marine spatial planning objectives are to identify risks to animals given their space use, usage including all activity types is required. However, specific events such as foraging can be prioritised for some applications (e.g. population consequences of disturbance; PCOD^28^), and under these circumstances, information in addition to usage maps would be required to fulfil conservation objectives.

Species distribution analyses often require underlying data to be aggregated into a static map^29^. The analysis presented here does not take patterns of residency and site turnover of animals into account. For example, mean usage does not differentiate between occasional use of an area by many individuals, or a small number of individuals utilising an area intensively. The number of individuals exposed to collision risk from marine renewable developments (e.g. tidal turbines) is likely to differ between these two situations. This is true of any static density inputs into collision risk models, and implications of not accounting for individual turnover in an area include predictions of collision risk that can exceed the total local population of animals, affecting the efficacy of the spatial management process^30^.

Spatial management can be informed through predicting movement of animals under given conditions, termed individual-based models (IBM)^31^. These models can be used to assess changes in species distributions over time and space, and as predictive tools to assess the impact of anthropogenic activities^32^. For central-place foragers in particular, predicting changes in distribution can be challenging. Central places can transition over time (e.g. seals can move to different haul outs, bats change roosting sites seasonally^33^), but the locations and time of switching to new central places can be difficult to predict. To provide more accurate analyses of changes in species distributions, environmental space can be parameterised within IBMs using underlying maps of habitat preference or space use^31^. These can provide information about the range of the species, areas of important habitat (e.g. optimal, sub-optimal, and unfeasible) to better inform movement and behaviour. For example, energetic costs of displacement when animals move from optimal to sub-optimal habitat due to anthropogenic activities can be quantified. High-resolution usage maps such as the ones presented here can be integrated within IBMs to produce a powerful analytical framework to predict change in species distributions to assess the impact of direct and indirect anthropogenic activities on protected species.

## Methods

### Study area

A study area centred on Orkney was delineated from 58.52°N to 59.66°N and 3.98°W to 1.88°W, to include the majority of telemetry data from the surrounding area (Fig. 4). To ensure that usage in the outer regions of the study area was not underestimated, a larger analytical area was delineated to capture telemetry data from animals that spent time at-sea within the study area. Emphasis was placed on determining a high grid resolution so that detailed space use could be represented. The underlying telemetry data were regularised to two-hourly intervals and the degree of kernel smoothing (see *Movement data*) to produce density surfaces was dependent on this regularisation. An appropriate spatial resolution of 0.6 km × 0.6 km was determined through estimation of median distance (median = 0.64 km; variance = 2.7 km) between each location of an individual. Analyses were conducted using R 3.3.2^34^ and GIS software Manifold 8.0.29.0^35^ and all maps were projected using Universal Transverse Mercator 30° North, World Geodetic System 1984 datum (UTM30N WGS84). Global Self-consistent, Hierarchical, High-resolution Geography Database (GSHHG) shoreline data version 2.2.2 from NOAA were used to represent land, available from http://www.soest.hawaii.edu/pwessel/gshhg/.

### Movement data

60 adult animals (defined as older than one year old), tagged between 2003 and 2015, spent time within the study area. Between 2003 and 2005, Satellite Relay Data Loggers (SRDL) were deployed that use the Argos satellite system for data transmission^36^. Between 2011 and 2015, GPS phone tags using the GSM mobile network with a Fastloc© hybrid protocol were deployed^37^. All animal handling procedures were approved by School of Biology, University of St Andrews Ethics Committee and carried out under Home Office Animals (Scientific Procedures) Act licence numbers 60/2589, 60/3303, 60/4009, and 60/7806. Telemetry data were processed through a set of data-cleansing protocols to remove observations with null and missing values, and duplicated records from the analysis.

SRDL positional error was corrected using a Kalman filter and data were used to estimate positions at two-hourly intervals^6, 38^. The majority of GPS locations have an expected error of ≤55 m^39^, although occasional outliers were excluded using thresholds of residual error and number of satellites, and then straight-line interpolated to regularise to the same two-hourly intervals as the SRDL data^6^. Three animals had few locations within the study area, and three animals did not have any haul out records, so these six animals were excluded, bringing the total number of animals used in the analysis to 54 (Table S1 and Fig. S1; Supplementary information).

Continuous spatial surfaces to represent the proportion of time animals spent in different areas were derived by kernel-smoothing the telemetry data. The *ks* R library^40^ was used to estimate spatial bandwidth of the 2D kernel applied to each animal/haul out site map. A multivariate plug-in bandwidth selector was determined for each individual by combining all locations associated with that individual. Individual-level weightings were applied to account for differences in the magnitude of data collected by an animal over its tag lifespan and for variation in the operational settings of the tag itself ^6^. This ensured that individuals with long tag lifespans, in which data could be highly auto-correlated, were not overrepresented, whilst also ensuring individuals with short tag lifespans were not underrepresented in the analysis. A discovery rate (termed *index of information content* ^6^) was determined as the total number of new grid cells that an individual visited during the tag lifespan. The *mgcv* library in R^41^ was used to fit a Generalised Additive Model (GAM) with a quasi-poisson distribution and a log-link function. The response variable was discovery rate and explanatory variables were the smooth of tag lifespan (hours) and tag type (SRDL or GPS) as a factor. Each animal/haul out map was multiplied by a normalised discovery rate and all density maps connected to each haul out cluster were aggregated and normalised to one.

### Terrestrial counts

**Figure 5:**
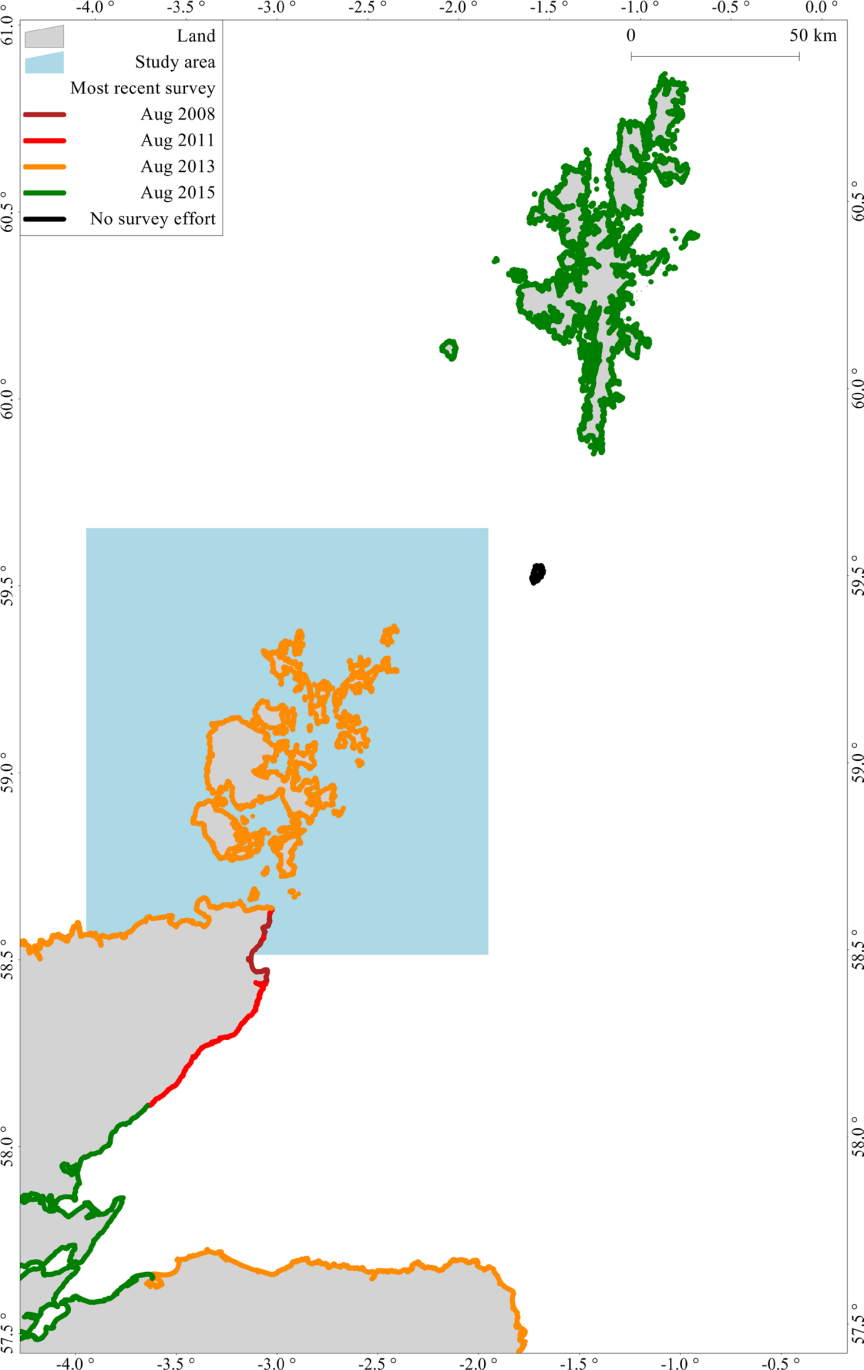
Map showing the most recent terrestrial surveys within the spatial extent of the analysis. Black lines represent ‘no survey effort’. The figure was produced using R 3.3.2^34^ and GIS software Manifold 8.0.29.0^35^.

Harbour seals are surveyed during their moult in August when the greatest number of animals haul out on land for an extended period. Different sections of coastline are surveyed each year. During aerial surveys all seals along a specified section of coastline are counted and coordinates are recorded to an accuracy of approximately 50 m. Surveys take place within two hours of low tide, when low tide is between 12:00 and 18:00 hours^42^. Surveyed coastline was gridded to 0.6 km × 0.6 km and the most recent available count (ranging from 2008 to 2015) was recorded in each onshore grid cell (Figs 5 and S2 in Supplementary information). Grid cells that were surveyed but in which no animals were located were given a value of zero. For each grid cell, the local population was estimated with associated uncertainty. Full details of this method are available from (Supplementary information www.int-res.com/articles/suppl/m534p235_supp.pdf6).

### Environmental data

1. **Shortest at-sea distance from haul out location**. By definition, central-place foragers have a home-range. For seals, this was represented by the shortest distance between a haul out site and an at-sea location taking into account land barriers (such as islands) that animals must swim around. Shortest at-sea distance was calculated using the *gdistance* R library^43^ at a resolution of 0.6 km × 0.6 km to determine distance between each seal location and the associated haul out (either departure or destination).
2. **Bathymetry**. The bathymetric metadata and Digital Terrain Model data products were derived from the European Marine Observation and Data Network (EMODNet) Bathymetry portal (http://www.emodnet-bathymetry.eu) released August/September 2015. Seabed depth data had a resolution of 1/8 minutes (about 230 m) and are based on the seabed depth at the Lowest Astronomical Tide (Fig. 6a).

**Figure 6:**
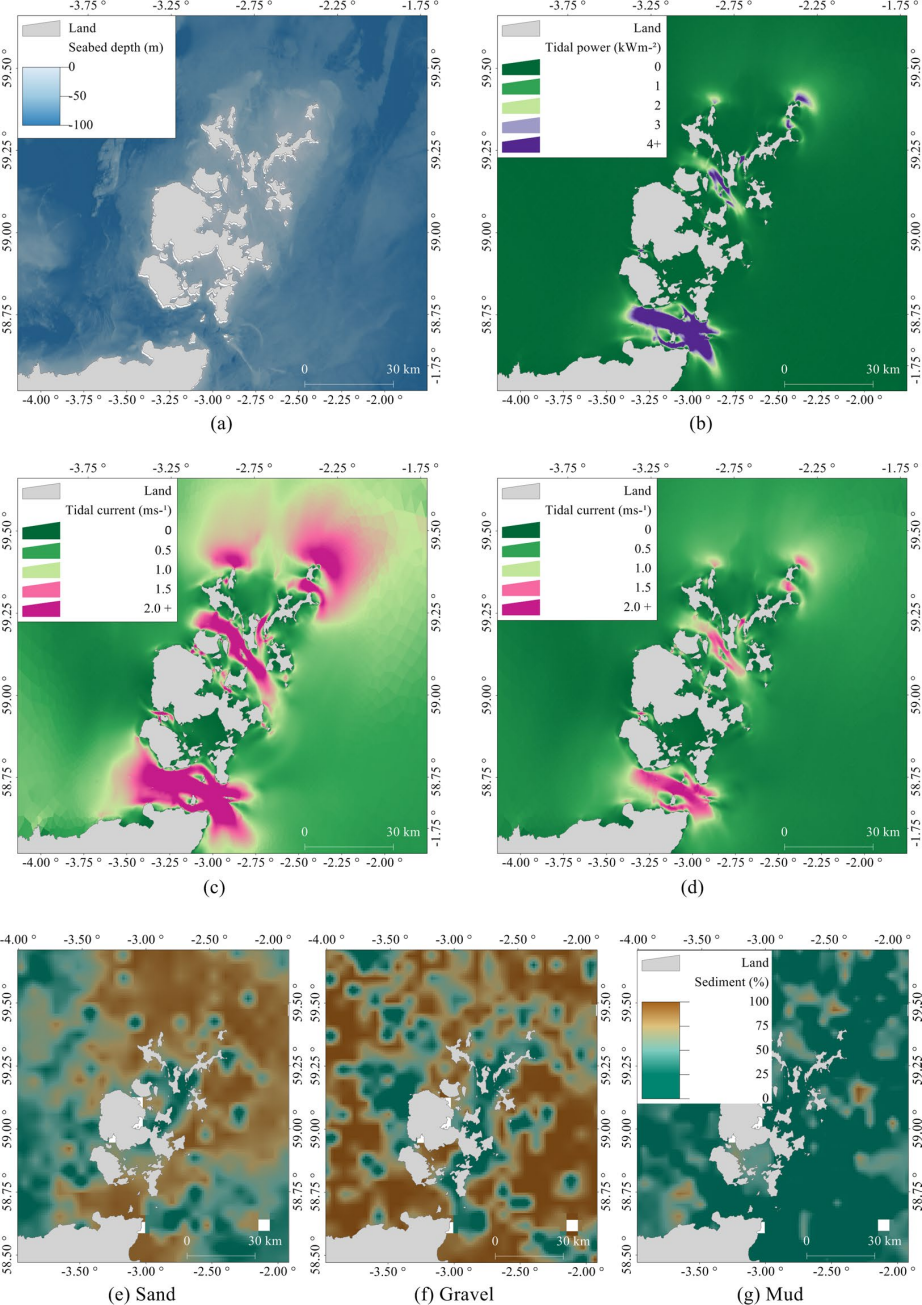
Environmental covariates (except shortest at-sea distance) used for habitat modelling: (**a**) Seabed depth, (**b**) Annual mean tidal power, (**c**) Peak flow for mean spring tide, (**d**) Peak flow for mean neap tide, (**e)** Proportion of sand in seabed sediment, (**f**) Proportion of gravel in seabed sediment, (**g**) Proportion of mud in seabed sediment. The figure was produced using R 3.3.2^34^ and GIS software Manifold 8.0.29.0^35^.
3. **Tidal power and peak flow**. Seals haul out on exposed areas of rock and sandbanks at low tide, and tidal information is likely to play an important role in their distribution^12^. Tidal energy resources were characterised by annual mean tidal power (kWm^−2^; Fig. 6b), peak flow for mean spring tide (ms^−1^; Fig. 6c), and peak flow for mean neap tide (ms^−1^; Fig. 6d), calculated using the Pentland Firth and Orkney Waters Hydrodynamic Model (PFOW)^10^. Mean peak current speeds were calculated using two tidal harmonics (M2; lunar and S2; solar) from 60 days of mid-depth velocity from the PFOW climatology run. The east and west components of current velocity were used to produce M2 and S2 amplitudes and phases. The semi-major axis amplitudes for each ellipse (M2 and S2) were then summed to produce peak flow for mean spring/neap tides^44^. To represent the kinetic energy available throughout the tidal cycle, annual mean tidal power ($\mathop{{P}_{T}}\limits^{\bar{} }$) was calculated. Average power available over 365 days from the PFOW climatology run was calculated taking a complete tidal cycle into account (rather than only peak values): $\mathop{{P}_{T}}\limits^{\bar{} }=1/2\rho \mathop{{U}^{3}}\limits^{\bar{} }$, where ρ is density of water, taken as 1027 kg m^−3^, and U is the mid-depth current speed^10, 44^. Model predictions were available in an unstructured grid ranging from a resolution of 150–250 m at the coast to 3 km at the outer edges of the study region.
4. **Sediment type** was derived from the British Geological Survey (available to download http://www.bgs.ac.uk), obtained from core samples spaced 5 km apart on average (Fig. 6e–g). A simplified Folk classification system^45^ was applied to derive variables containing proportions of sand, gravel, and mud. Data were given as a percentage-by-weight of gravel (particles > 2.0 mm in diameter), sand (0.0625–2.0 mm in diameter), and mud (particles < 0.0625 mm in diameter). Spatial autocorrelation between the three covariates was calculated by randomly sub-sampling the cores to calculate semi-variograms^46^. Each sediment covariate was kriged at a 1 km resolution using the semi-variograms and the resultant local estimates were normalised^17^. These covariates did not account for other substrate (such as underlying rock or biotope information) that may have been present on the seabed.

### Haul out clustering

A 0.6 km × 0.6 km grid cell was identified as an onshore haul out either from the telemetry data where animals moved onto land, or from the terrestrial count data where animals were counted within that cell. Haul out cells were aggregated for the purpose of scaling to a local population level because: (a) The resolution of a 0.6 km × 0.6 km cell may not have been consistent with the scale of animal behaviour and space use if more than one haul out formed part of a connected aggregation (e.g. seals may return to an onshore location close to departure haul out); (b) using non-aggregated haul outs maximised the number of haul out cells defined by the terrestrial count data that did not have telemetry data directly associated with them. This would have resulted in inflated uncertainty as the habitat model would contribute more usage to the analysis than necessary; and (c) using non-aggregated haul out cells associated with telemetry data but where the terrestrial count was zero reduced the importance of telemetry data (effectively removing telemetry data from the usage surface). Haul out cells were aggregated using a clustering algorithm based on shortest at-sea distance between them. To define an appropriate spatial scale, hierarchal cluster analysis with a centroid agglomeration method was used to generate clustering ranges from a minimum separation of 0.6 km (no clustering) to 15 km (maximum clustering) in increments of 0.6 km^47^. A change point analysis was performed based on the number of clusters using the *changepoint* R library^48^. A single change point occurred at 3.6 km and haul outs were aggregated to this scale for the remainder of the analysis. *Telemetry clusters* were defined as having telemetry data from at least one tagged animal associated with any haul out cell in the cluster. *Null clusters* were those where terrestrial count data showed seals were present, but no tagged animals visited any haul out cells within the cluster. To retain telemetry clusters with zero terrestrial counts in the analysis, their counts were changed to one, and the total was rescaled to the original count.

### Habitat modelling

Predictions of at-sea usage were required for null clusters (where seals were known to haul out from the terrestrial count data but for which no telemetry data were available). Augmenting the approach taken in Jones *et al*. (2015), a Generalised Additive Modelling – Generalised Estimating Equation (GAM-GEE) modelling framework was used to predict at-sea seal usage. Models were fitted using all telemetry locations with five pseudo-absences associated with each presence point by repeatedly selecting at-sea locations within the study area to associate a representative range of underlying environmental covariates with the pseudo-absence points^49^. Multicollinearity between the covariates was tested using Variation Inflation Factor (VIF) analysis from the *car* R library^50^. Peak flows for mean spring and neap tides were highly correlated (based on a threshold for high collinearity > 5) so these covariates were not included in the same model during model selection. All other covariates had a VIF score between 1.5 and 3.7. The *geepack* R library^51^ was used to fit binomial GAM-GEEs with a logit link function and an independent working correlation structure to account for any residual autocorrelation within defined panels of data^52^. Panels were defined for individual animal and for pseudo-absences separately to avoid underestimating autocorrelation within presences of an individual, and each pseudo-absence was assumed to be independent^26^. Covariates were standardised (mean = 0, sd = 1) to aid model fitting^53^. Year of tag deployment was included as a factor and shortest at-sea distance was included as a linear covariate within the linear predictor. The *splines* R library was used to implement cubic *β*-splines to allow all other covariates to vary as a function of one-dimensional smooth terms within the linear predictor (4 degrees of freedom) with one internally positioned knot at the mean of each covariate^52^. Linear and spline terms were offered in model selection for all covariates. Allowing interactions between covariates was not possible due to non-convergence in the models. Models were assessed on their ability to predict spatially, and similar-sized spatial blocks were delineated based on haul out cluster using the *sample* function in R. Forwards model selection was carried out using *k*-fold cross-validation, using four blocks to fit a model and predicting from the fifth block. This was repeated five times until all blocks had been used in prediction. For each fold, equal-areas with 40 bins with a moving window were used and Spearman rank correlations were calculated based on *n* = 40 and α = 0.05. Folds passing this test were summed and the count divided by five. The threshold for fold pass score (FPS) for five-folds was FPS > 0.8^54^.

The selected model was used to estimate usage for the study area for each null cluster. The median value for tag deployment year (2011) was used for all predictions and shortest at-sea path from haul out cluster was calculated. Predicted (mean population) space use was calculated from the exponential of the linear predictor^17^. For each null cluster, space use was normalised to one, so that it could be scaled to the local population estimate.

### Propagating uncertainty and population-scaling

Uncertainty within each grid cell of the usage maps was calculated. Within-cluster variance was modelled using data-rich telemetry clusters (determined experimentally to be those sites which had ≥ 7 tagged animals associated with them). Variance was estimated from linear models with explanatory covariates of sample size (number of tagged animals in the telemetry cluster) and mean usage by seals. The models predicted variance for data-poor telemetry and null clusters (by setting the sample size of the uncertainty model to zero). Predicted within-cluster variance increased as the mean usage and number of tagged animals decreased (Supplementary information Fig. S4). The harbour seal population in each cluster was estimated from terrestrial count data, which were rescaled to allow for the proportion of animals at sea when surveys were carried out^55^. Population-level variance for each cluster was calculated from bootstrapping, based on the uncertainty in estimates of haul out probability^6^. Within-cluster and population-level variances were combined to give uncertainty estimates for each grid cell in the usage maps. Maps for all clusters were then scaled according to the local harbour seal population, also accounting for the mean proportion of time animals spent at sea (calculated from the telemetry data). Density estimation maps (using telemetry data) were combined with habitat model predictions of usage for null clusters to create total usage maps, showing mean usage with associated 95% confidence intervals.

### Data availability

The datasets analysed during the current study are available in the Pure repository, http://dx.doi.org/10.17630/4f86d1c0-f999-4ca2-b6a8-6ea63a83400b.

## Electronic supplementary material

Supplementary information
