# Supplementary material for: Fine-scale harbour seal usage for informed marine spatial planning
Source: Sci Rep. 2017 Sep 14;7:11581. doi: 10.1038/s41598-017-11174-4 (PMC5599608; doi:10.1038/s41598-017-11174-4)
Supplement: Supplementary file 1 — Supplementary information [file 41598_2017_11174_MOESM1_ESM.pdf]

Fine-scale harbour seal usage for informed marine spatial planning

Esther L. Jones*[1,2], Carol E. Sparling (ces@smruconsulting.com)[3], Bernie J. McConnell[2] (bm8@st-andrews.ac.uk), Christopher D. Morris[2] (cdm21@st-andrews.ac.uk), Sophie Smout[2,1] (scs10@st-andrews.ac.uk)

Supplementary information

Table S1. Animals included in the analysis showing animal reference number, tag type, age-class, region, location of tagging, sex, year tagged, and number of days of movement data collected.

#	Animal reference	Tag type	Age	Management region	Tagging location	Sex	Year	Tagged days
1	pv1-ali-03	SRDL	1+	Orkney & N coast	Sanday	F	2003	187
2	pv1-Arnie-03	SRDL	1+	Orkney & N coast	Eynhallow	M	2003	179
3	pv1-bo-03	SRDL	1+	Orkney & N coast	Sanday	F	2003	184
4	pv1-Bob-03	SRDL	1+	Orkney & N coast	Eynhallow	M	2003	131
5	pv1-cat-03	SRDL	1+	Orkney & N coast	Sanday	F	2003	217
6	pv1-dot-03	SRDL	1+	Orkney & N coast	Sanday	F	2003	273
7	pv1-erin-03	SRDL	1+	Orkney & N coast	Rousay	F	2003	165
8	pv6-Ken-04	SRDL	1+	Orkney & N coast	Stronsay	M	2004	143
9	pv6-Len-04	SRDL	1+	Orkney & N coast	Stronsay	M	2004	112
10	pv6-Max-04	SRDL	1+	Orkney & N coast	Rousay	M	2004	78
11	pv6-Oli-04	SRDL	1+	Orkney & N coast	Eynhallow	M	2004	92
12	pv6-pat-04	SRDL	1+	Orkney & N coast	Stronsay	F	2004	115
13	pv6-Pete-04	SRDL	1+	Orkney & N coast	Eynhallow	M	2004	26
14	pv6-queenie-04	SRDL	1+	Orkney & N coast	Rousay	F	2004	99
15	pv6-sally-04	SRDL	1+	Orkney & N coast	Eynhallow	F	2004	74
16	pv11-James-05	SRDL	1+	Moray Firth	Dornoch	M	2005	32
17	pv11-Kath-05	SRDL	1+	Moray Firth	Dornoch	F	2005	108
18	pv24-112-11	GPS	1+	Orkney & N coast	Pentland	M	2011	167
19	pv24-148-11	GPS	1+	Orkney & N coast	Pentland	M	2011	143

20	pv24-150-11	GPS	1+	Orkney & N coast	Pentland	F	2011	113
21	pv24-151-11	GPS	1+	Orkney & N coast	Pentland	M	2011	72
22	pv24-153-11	GPS	1+	Orkney & N coast	Pentland	F	2011	121
23	pv24-155-11	GPS	1+	Orkney & N coast	Pentland	M	2011	36
24	pv24-165-11	GPS	1+	Orkney & N coast	Pentland	M	2011	48
25	pv24-394-11	GPS	1+	Orkney & N coast	Pentland	M	2011	88
26	pv24-541-11	GPS	1+	Orkney & N coast	Pentland	M	2011	133
27	pv24-580-11	GPS	1+	Orkney & N coast	Pentland	F	2011	94
28	pv24-590-11	GPS	1+	Orkney & N coast	Pentland	M	2011	71
29	pv24-598-11	GPS	1+	Orkney & N coast	Pentland	F	2011	110
30	pv24-622-11	GPS	1+	Orkney & N coast	Pentland	M	2011	76
31	pv24-x625-11	GPS	1+	Orkney & N coast	Pentland	M	2011	84
32	pv44-003-12	GPS	1+	Orkney & N coast	Eday	F	2012	41
33	pv44-004-12	GPS	1+	Orkney & N coast	Eday	F	2012	41
34	pv44-005-12	GPS	1+	Orkney & N coast	Eynhallow	M	2012	51
35	pv44-007-12	GPS	1+	Orkney & N coast	Eday	F	2012	40
36	pv44-011-12	GPS	1+	Orkney & N coast	Eynhallow	M	2012	51
37	pv44-014-12	GPS	1+	Orkney & N coast	Eynhallow	M	2012	44
38	pv44-017-12	GPS	1+	Orkney & N coast	Eday	M	2012	41
39	pv44-018-12	GPS	1+	Orkney & N coast	Eday	M	2012	26
40	pv44-020-12	GPS	1+	Orkney & N coast	Eday	F	2012	32
41	pv44-021-12	GPS	1+	Orkney & N coast	Eday	F	2012	25
42	pv47-392-12	GPS	1+	Orkney & N coast	Eynhallow	M	2012	110
43	pv47-427-12	GPS	1+	Orkney & N coast	Eynhallow	M	2012	17
44	pv47-539-12	GPS	1+	Orkney & N coast	Eday	M	2012	143
45	pv47-583-12	GPS	1+	Orkney & N coast	Eynhallow	M	2012	99
46	pv47-585-12	GPS	1+	Orkney & N coast	Eday	M	2012	151
47	pv47-588-12	GPS	1+	Orkney & N coast	Eynhallow	M	2012	93
48	pv57-197-14	GPS	1+	Orkney & N Coast	St Margarets	F	2014	88
49	pv57-199-14	GPS	1+	Orkney & N Coast	Switha	M	2014	5
50	pv57-200-14	GPS	1+	Orkney & N Coast	St Margarets	F	2014	151
51	pv57-913-14	GPS	1+	Orkney & N Coast	St Margarets	F	2014	176
52	pv59-05-15	GPS	1+	Moray Firth	Loch Fleet	F	2015	121
53	pv59-07-15	GPS	1+	Moray Firth	Loch Fleet	F	2015	141
54	pv59-12-15	GPS	1+	Moray Firth	Loch Fleet	F	2015	128

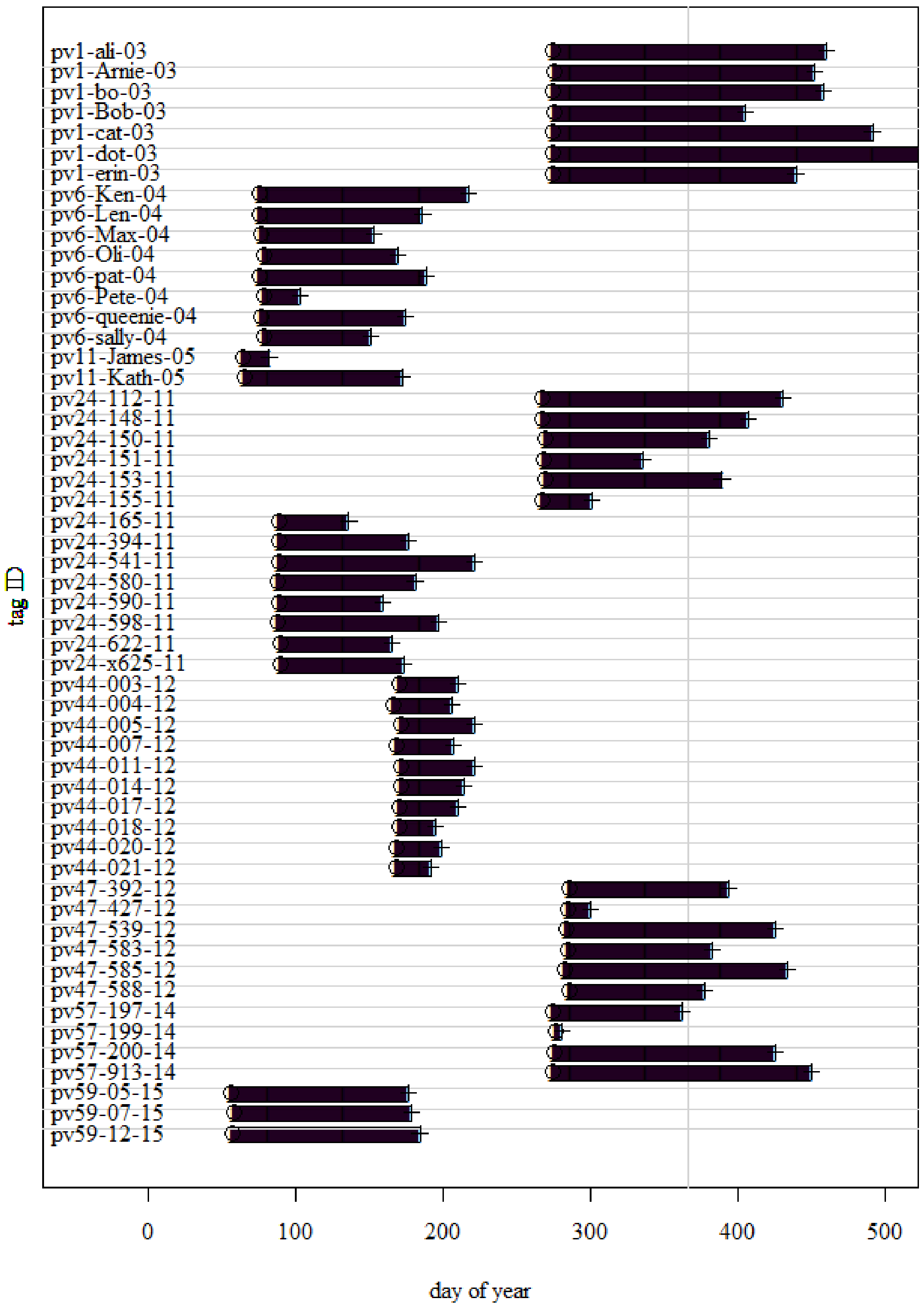

Figure S1. Temporal extent (day of year) of movement data by animal.

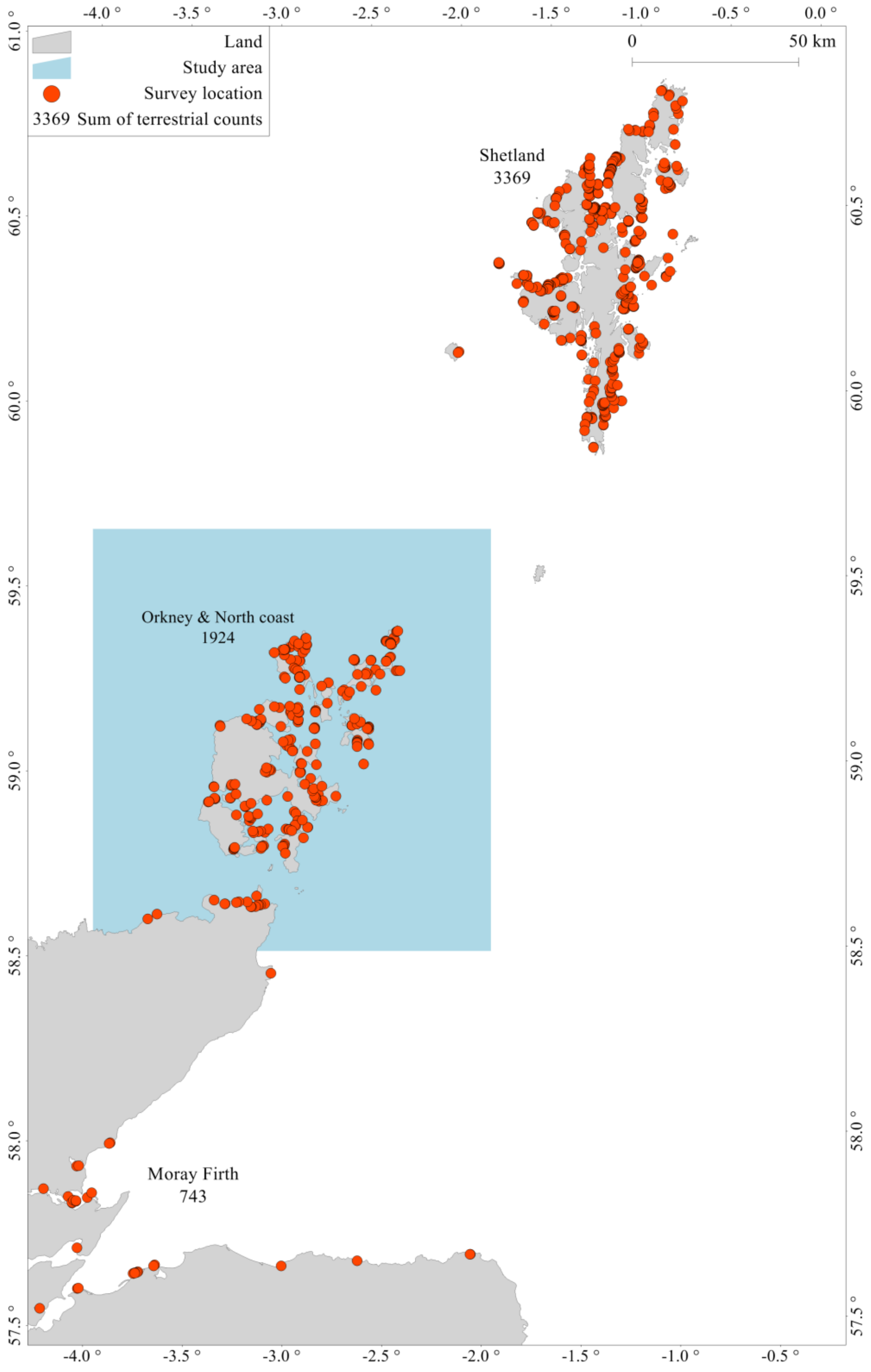

Figure S2. Locations and totals by regions of the most recent terrestrial survey counts (ranging from 2008 to 2015 for individual locations) within the spatial extent of the analysis. The figure was produced using R 3.3.2[34] and GIS software Manifold 8.0.29.0[35].

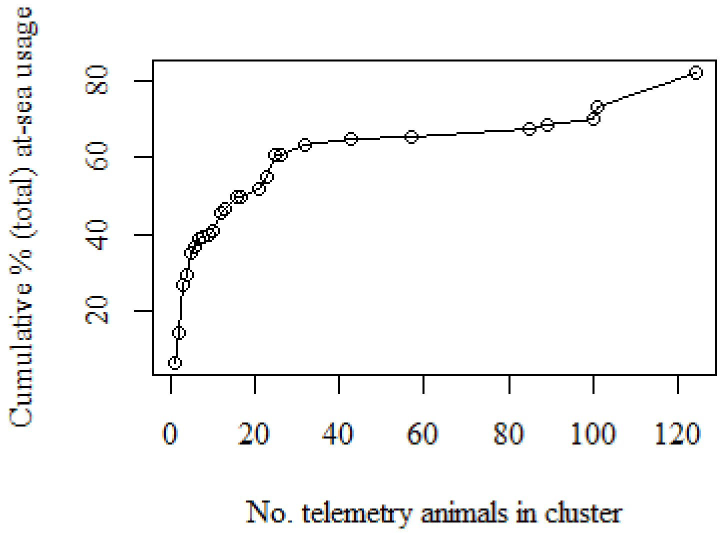

Figure S 3. Cumulative usage contribution that haul out clusters with increasing numbers of tagged animals make to the total mean at-sea usage.

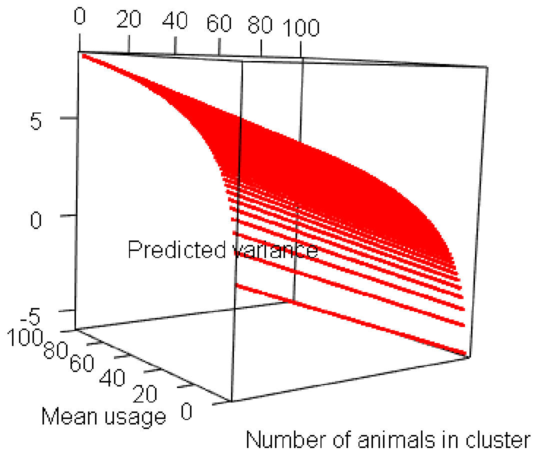

Figure S 4. Predicted within-cluster variance when mean density and number of animals in a haul out cluster are varied from 1 to 100 respectively.
